# Are Smart Homes Adequate for Older Adults with Dementia?

**DOI:** 10.3390/s22114254

**Published:** 2022-06-02

**Authors:** Gibson Chimamiwa, Alberto Giaretta, Marjan Alirezaie, Federico Pecora, Amy Loutfi

**Affiliations:** Centre for Applied Autonomous Sensor Systems (AASS), Örebro University, 70281 Örebro, Sweden; alberto.giaretta@oru.se (A.G.); marjan.alirezaie@oru.se (M.A.); federico.pecora@oru.se (F.P.); amy.loutfi@oru.se (A.L.)

**Keywords:** smart homes, ageing, dementia, activity recognition, habit recognition

## Abstract

Smart home technologies can enable older adults, including those with dementia, to live more independently in their homes for a longer time. Activity recognition, in combination with anomaly detection, has shown the potential to recognise users’ daily activities and detect deviations. However, activity recognition and anomaly detection are not sufficient, as they lack the capacity to capture the progression of patients’ habits across the different stages of dementia. To achieve this, smart homes should be enabled to recognise patients’ habits and changes in habits, including the loss of some habits. In this study, we first present an overview of the stages that characterise dementia, alongside real-world personas that depict users’ behaviours at each stage. Then, we survey the state of the art on activity recognition in smart homes for older adults with dementia, including the literature that combines activity recognition and anomaly detection. We categorise the literature based on goals, stages of dementia, and targeted users. Finally, we justify the necessity for habit recognition in smart homes for older adults with dementia, and we discuss the research challenges related to its implementation.

## 1. Introduction

Dementia is one of the leading causes of disability and dependency among the ageing population [1]. It affects an estimated 50 million people worldwide, with 10 million new cases reported each year. In 2019, the global societal cost of dementia care was estimated at USD 1.3 trillion and is projected to increase to USD 1.7 trillion by 2030 [2]. With the aim of delaying the institutionalisation of patients, many countries have invested in shifting the focus from institutional care to community care provided by informal family carers [3], known as the *sandwich generation* [4]. Although this strategy may allow older adults to live longer in their preferred environments, at a lower cost, it overburdens the caregivers as the health condition of patients worsen. The rise in the use of technology to support older adults, including those with dementia, could serve to reduce this burden [5].

Smart homes are ambient intelligent environments with the capability to assist users in performing their daily activities [6]. Smart homes are equipped with interconnected software and hardware components to monitor and recognise the activities that are performed by the home’s occupants. The process of activity recognition (AR) in smart homes involves installing sensors to capture activities and changes in the environment’s state, processing the sensor data, creating activity models, and inferring activities from sensor events [7]. By analysing the sensor data through techniques such as machine learning models [8], the activity patterns can be recognised. Furthermore, anomalies which indicate deviations from normal activity patterns can be captured. An anomaly represents a pattern in data that deviates from the expected behaviour, and the process of discovering such patterns is referred to as anomaly detection (AnD) [9]. Such anomalies are either communicated as reminders for the user to perform, e.g., forgotten activities, or as alerts for caregivers to provide further assistance [10]. In this way, smart homes can enhance the possibility of older adults to live independently while feeling safe [11,12].

In a recent study, Moyle et al. [13] investigated the effectiveness of smart home technologies to support the health outcomes of older adults with dementia (OAwDs) residing in community-based homes. The authors assessed five smart home systems in terms of residents’ physical activity, activities of daily living, sleep, anxiety, depression, agitation, irritability, risk of falls, cognitive functioning, night-time injury, and exiting their homes unattended. They concluded that current smart homes are not sufficient for supporting dementia patients as they lack thorough evaluation.

### 1.1. Contributions

In this paper, we present the technical capabilities of smart homes for OAwDs and then discuss why existing solutions are not sufficient for dementia patients, as argued by Moyle et al. [13]. Our contributions are manifold:

- First, we survey the literature and map the existing works on activity recognition and anomaly detection for OAwDs to the various stages of dementia, based on the cognitive health of the users.
- We point out that activity recognition and anomaly detection lack the capability of detecting transitions between stages of dementia. Using personas proposed in the literature, we justify the necessity of habit recognition for detecting such transitions.
- Finally, we present some research challenges that need to be addressed in order to implement habit recognition in smart homes for OAwDs.

### 1.2. Outline

This paper is structured as follows. In Section 2, we present an overview of the different stages of dementia and present some realistic personas reflecting the experiences of OAwDs at each stage. In Section 3, we briefly discuss the current state of smart homes. We survey the literature on activity recognition and a combination of activity recognition and anomaly detection in smart homes for OAwDs in Section 4, and categorise the methods based on the goal, the stages of dementia, and the targeted users. In Section 5, we survey the literature on habit recognition and argue why the current approaches are not sufficient to capture the habits and changes in habits of OAwDs. We present our conclusions in Section 7.

## 2. Dementia

Dementia is a progressive decline in memory and cognitive function that affects the ability to perform daily activities independently [14]. It affects memory, the ability to think, orientation, comprehension, calculation, learning ability, language, and judgement. The most common type of dementia is Alzheimer’s disease (AD), which accounts for 60–70% of cases. Although no treatment exists for dementia, it is possible to support and improve the patients’ quality of life [1]. Early diagnosis provides the opportunity for early and optimal management, which could slow the progression of OAwDs to later stages of dementia. Other support measures include maintaining physical health, cognition, activity, well-being, and managing behaviour changes.

**Figure 1:**
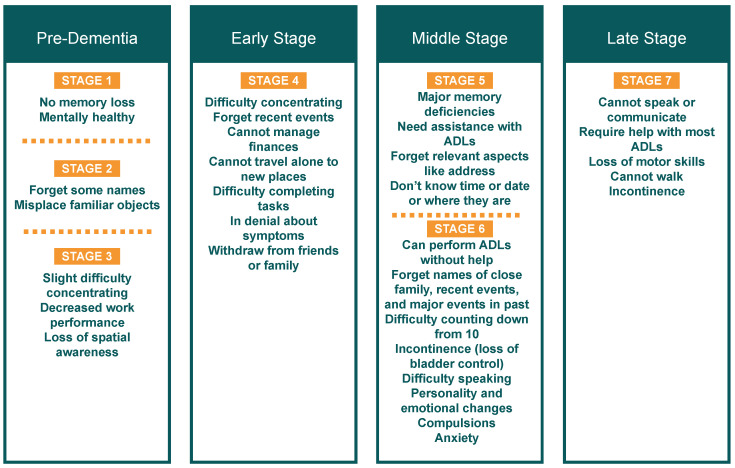
Stages of Dementia (based on the GDS [15]).

The Global Deterioration Scale for the Assessment of Primary Degenerative Dementia (GDS) [15] is commonly used for measuring the progression of dementia. The scale categorises the symptoms of dementia into seven stages, which are further combined into four main stages, namely, the pre-dementia stage, early stage, middle stage, and late stage. Figure 1 shows examples of behavioural characteristics associated with each stage of dementia based on the GDS.

Individual symptoms of dementia at each stage may vary from person to person, depending on other health conditions [1]. This creates a challenge in the provision of care and other support needs. Personas are design tools that help to elicit users’ individual characteristics to develop solutions tailored for their specific needs [16]. With respect to dementia, personas may be effective tools to understand each patient’s symptoms, challenges, and abilities at the different stages. In the Ecare@Home [17] project, several personas were developed for different use cases to highlight the need for smart home technologies to address specific user requirements in their homes.

Here we present a detailed description of the symptoms at each stage of dementia, together with personas which describe user behaviours matching the symptoms. The personas, which are taken from the literature [18,19,20], help to illustrate the progression of patients at each stage. Furthermore, we use one of the personas to justify the need for habit recognition in Section 5.

**Pre-dementia stage**: GDS stages 1, 2, and 3 fall under the pre-dementia stage, characterised by subjective feelings of memory decline such as misplacing items, forgetting names, and getting lost in familiar environments. Although there are no clinical characteristics of dementia at these stages, distinct signs of cognitive deficit begin to manifest themselves at stage 3, also known as mild cognitive impairment (MCI). This phase is critical for detecting the early signs of dementia, as it provides an opportunity to make proactive interventions to slow the progression to later stages [21].

Persona [18]: *A.V. is a 74-year-old Caucasian woman with a diagnosis of aMCI (amnestic MCI) and mild depression. Over the past few years she has started to experience visible impairment in performing the activities of daily living. Her family has also observed a progressive decline in her cognitive ability in the last 2 years, including poor memory, notably for conversation and instructions, and sometimes disorientation. During this period, Mrs. A.V’s anxiety also worsened and her emotional reactions and behaviour became erratic, as she began to forget things regularly. She would miss appointments with friends or forget special events (e.g., family birthdays) and names. Mrs. A.V. had increased difficulties with personal hygiene and keeping up with housework due to exhaustion and forgetting tasks. For instance, when she started cleaning her house, she would stop after an hour due to tiredness. Eventually, her social interactions also decreased.*

**Early stage**: Stage 4 represents the early stage of dementia, with clear-cut clinical characteristics associated with the disease. Some of the symptoms that emerge at this stage include difficulties in remembering recent events or one’s personal past, reduced ability to travel, and an inability to handle personal finances. Most patients at this stage live in denial about their memory or other cognitive changes.

Persona [18]: *P.K. is an 80-year-old Caucasian man with a diagnosis of mild dementia. He experienced complex physical and cognitive impairment in performing activities of daily living, e.g., managing his finances. He lived alone for 10 years. Mr P.K. had a 3 year record of gradual memory problems, which began about 5 years after the death of his wife. His son and his daughter noticed mild problems regarding activities of daily living, including forgetting to pay bills and taking medicine. Mr P.K. denies any symptom of depression, although he admitted to the clinician that sometimes he cried with no specific reason.*

**Middle stage**: Stages 5 and 6 form the middle stage of dementia, characterised by an increased need of support to perform daily activities. Patients may struggle to remember personal information such as addresses or phone numbers, forget names of close relatives, and become disoriented. Individuals may repeatedly perform simple tasks (e.g., cleaning), become more anxious and agitated, and lose their diurnal rhythm.

The persona [19]: *Frida is an 82 year old woman who was diagnosed with Alzheimer’s five years ago (MMSE = 10). After taking a nap in the afternoon, she is watching TV in her living room with Rosa, a retired nurse who cares for her during the day. Suddenly, Frida walks to her room and starts taking clothes out of her closet. Rosa comes in, realizes that she is putting her clothes in a suitcase, and asks Frida why she is doing this. Frida replies that it is getting late and she needs to get back home. Rosa tells her that she is currently at her house, but Frida insists that her home is in another city, where she used to live 30 years ago. After several minutes discussing the issue, Rosa convinces Frida to stop trying to leave home by showing her some family photographs.*

**Late stage**: Stage 7 is the late stage of dementia, characterised by the patient’s loss of significant verbal ability and psychomotor skills. At this stage the patient requires assistance to perform all daily activities, including feeding, grooming, walking, and toileting.

Persona [20]: *Susan is an 88-year-old widow, who enjoyed cooking, knitting, and talking about politics. She has poor eyesight and hearing, and she gets distressed by noises. She is susceptible to smelling and touching and she is afraid of the dark. Susan has a limited ability to communicate verbally in a cohesive way. Often, she has aggressive outbursts and moans constantly, and periodically throws objects at carers. She isolates herself and suffers from delusions. Susan depends on family members and carers to make decisions. She is also unable to recognise family members and friends or to process things, and is continuously confused with her environment. She is dependent on assistance and walking aids to walk very short distances. Susan has difficulties with feeding herself and swallowing food, and is also suffering from incontinence.*

The various stages reveal the fine-grained behaviour characteristics of patients as the disease progresses. To support OAwDs, caregivers and medical professionals need to match the observable behaviours of the patient to the symptoms. However, this process is not trivial, as there are many overlapping characteristics between some stages [22]. The personas show that, as dementia progresses along different stages, it becomes more difficult for users to accomplish their daily activities independently. This loss of independence poses health and security risks, such as falls or getting lost due to wandering [23]. Due to these challenges, research on assistive technologies for dementia in home environments is gaining momentum.

## 3. Smart Homes for Older Adults with Dementia

As previously mentioned in Section 1, smart homes are ambient assisted-living environments equipped with smart technologies (both hardware and software) to assist users in performing their daily activities [24]. Sensor devices play a crucial role in smart homes, allowing the continuous monitoring of occupants’ daily activities. Smart home sensors fall under three main categories: ambient sensors, wearable sensors, and visual sensors [25,26]. Ambient sensors, such as passive infrared sensors (PIR), pressure sensors attached to the bed or couch, and contact switch sensors (CSS) attached to doors or closets, can capture activities such as walking, sleeping, or exiting the house, respectively. Wearable sensors (accelerometers, gyroscopes, wrist watches, and heart rate sensors) can monitor users’ physical activities and physiological measurements. Visual sensors, such as video cameras, can capture and record different activities performed by users and provide accurate insights. In the context of dementia, smart home technologies can be used to monitor and support the patients as they accomplish their daily activities, thereby allowing them to live more independently and safely for as long as possible [27]. In their study, to determine the requirements of smart homes for dementia patients, Orpwood et al. [28] state that monitoring sensors should be non-intrusive, to reduce confusion and anxiety in the patients. Ambient sensors are non-intrusive and effective for detecting activities [26], as they are easy to deploy in homes and guarantee users more safety and privacy than wearable and visual sensors.

Wearable sensors can allow the monitoring of users in accomplishing their activities, but they can also cause discomfort for people with physical or cognitive challenges, such as OAwDs. Finally, although visual sensors have the disadvantage of being privacy-invasive, they can capture important events, such as falling incidents, which are prevalent among OAwDs [29].

Smart homes not only make OAwDs feel safe, but can also assess and assist their cognitive and functional health [10]. Orpwood et al. [28] state that prompts and reminders are a requirement for smart homes for dementia patients to provide the required interventions. Users in the later stages of dementia may struggle to complete complex activities that require them to execute tasks in sequence. For example, washing clothes involves executing several actions, e.g., putting clothes into the washing machine, adding soap, and starting the machine [30]. While executing this sequence of tasks, a dementia patient may start the washing machine but forget to first load the dirty clothes. By combining different sensor events and reasoning about the sequence, anomalies can be captured. In the case of washing clothes, a motion sensor could capture the movement of the user around the washing machine, a contact sensor could detect when the washing machine door is opened and closed, and a current sensor could detect when the washing machine is in use. If a user starts the washing machine without first opening and closing the door, the smart home system may trigger an alert to remind the user to load the washing machine.

In the next section, we provide a comprehensive overview of the state of the art in relation to activity recognition and anomaly detection techniques for OAwDs in smart homes.

## 4. Activity Recognition in Smart Homes for Older Adults with Dementia

Activity recognition in smart homes for OAwDs has been widely investigated over the past few decades. In this section, we discuss activity recognition in smart homes for OAwDs and summarise the methods based on the stages of dementia presented in Section 2. In cases where the studies do not explicitly state the targeted dementia phase, we determine the stage by combining the goal and the targeted users. For instance, studies that monitor and analyse the activities of healthy or MCI patients fall under the pre-dementia stage, as the users are not yet clinically diagnosed with dementia. Similarly, we categorise methods that involve dementia patients under the category of early-to-middle stage dementia, as no distinct stage is specified. Of the studies investigated, none focus on specific characteristics of OAwDs in late-stage dementia. This stems from the fact that most patients at this stage are completely dependent on human support to perform daily activities. Table 1 shows a summary of the activity recognition methods for OAwDs.

It is worth noting that, although some of the studies discussed in this section focus solely on activity recognition, the majority combine activity recognition with anomaly detection to capture abnormal activities that indicate cognitive impairment. Therefore, in this study, we make the assumption that anomaly detection is an extra layer on top of activity recognition. Common smart home datasets used for activity recognition include CASAS [31], UCI [32], van Kastaren [33], ORCATECH [34], Dem@Care [35], TIHM [36], and Ecare@Home [37]. Of these datasets, only Dem@Care, ORCATECH, and TIHM are focused on the activities of dementia patients, whereas the rest are targeted at healthy users.

### 4.1. Activity Recognition for the Pre-Dementia Stage

In this section, we discuss activity recognition methods targeting healthy older adults (HOAs) and MCI patients. Users at this stage are not yet clinically diagnosed with dementia but may show signs of forgetfulness, as indicated on the GDS. To address such challenges, some AR methods in smart homes analyse sensor data to assist users in performing their daily activities. For instance, Feuz et al. [38] proposed a method to recognise users’ activity transitions and trigger reminders for MCI patients to complete their activities. The authors used a decision tree (DT) to map sequences of sensor events to activity labels, and to learn activity transitions. By detecting changes in the sensor data through probability distribution changes, a change-point detection was obtained. The results showed the potential to detect activity transitions but was not integrated in a real-time prompting system.

Other activity recognition methods have focused on capturing behavioural differences between HOAs and users with cognitive impairment. Cook et al. [39] used activities and movement patterns to distinguish HOAs from MCI patients. The authors classified features (activity duration, mean, and standard deviation) using machine learning algorithms (support vector machine (SVM), DT, naïve Bayes (NB), random forest (RF), and adaptive boosting (AdaBoost)) to identify the groups. With enough data, the approach showed the potential to distinguish HOAs from MCI patients, detect signs of cognitive impairment, and enable early intervention.

König et al. [40] also captured behavioural differences based on the number of completed activities and the correct sequence of task execution. The authors applied video analysis to extract kinematic parameters of activity performance, and contextual information (the user’s location, posture, and proximity to an object). The method was compared with human observation and assessment scores and was able to distinguish between HOA and MCI patients. Jekel et al. [41] also analysed video data in combination with sensor data to detect behavioural differences between the same user groups. Non-parametric tests were used for the analysis as the data lacked a normal distribution. The analysis results showed that patients with MCI had more difficulties in performing activities and in their correct sequence than HOAs.

Still with the same focus, Akl et al. [42] distinguished between HOAs, unknown, and MCI patients based on the predefined walking speed and general activity of users. The authors applied RF and SVM techniques to classify several features such as the average and probability density of the walking speedto identify the groups. Their results showed that the approach can be used to identify the users’ behavioural differences. Similarly, Javed et al. [43] proposed a method that captures behavioural differences by considering predefined scores from a neuropsychologist, to quantify users’ activity performance. The authors used simple features and complex features to train the Cognitive Assessment of Smart Home Residents (CA-SHR) classifier, which showed the potential to predict the cognitive status of HOAs and MCI, and dementia patients. The model demonstrated better classification accuracy in comparison to other techniques such as NB and Adaboost.

Instead of analysing sensor or video data to capture activities, Kaye et al. [44] used computer-use data to differentiate HOAs from MCI patients. The authors analysed mouse movements captured by computer tracking software, whereas the sensors only monitored the user’s presence at home. They applied linear mixed effects models to compare the users, and the results showed a decline in the total and mean daily use, as well as an increase in the daily variability of use among MCI patients relative to HOA.

Another goal of activity recognition in smart homes is to analyse activity sensor data to capture individual behavioural changes, which may indicate the patient’s cognitive status. For instance, Hussain et al. [45] investigated a method of learning the activity patterns of HOAs and MCI patients and of detecting behavioural changes associated with AD. The authors applied a generating alternative task sequence (GATS) algorithm to extract simple tasks and map them to high-level activities using different classifiers, such as SVM, NB, and K-nearest neighbour (KNN). The models performed better at recognising activities and changes in behaviour compared to traditional techniques. Similarly, Gochoo et al. [46] proposed a method to analyse the indoor travel patterns of elderly people to detect wandering behaviour based on the Martino–Saltzman (MS) model [76]. They classified features (the number of movements, their duration, and repeated locations) using a deep convolutional neural network (DCNN) to capture the travel patterns. The classifier performed well on the MS model but was not evaluated for dementia wandering due to the lack of patient data and annotations.

### 4.2. Anomaly Detection for the Pre-Dementia Stage

The methods discussed in this section focus on anomaly detection to accomplish different objectives such as assisting users with task completion, capturing behavioural differences between HOAs and MCI patients, detecting early signs of dementia, and providing intelligent reasoning and automated health assessments.

Similarly to activity recognition, as discussed in the previous section, the detection of anomalies allows smart home systems to assist users in completing activities or assist caregivers to support users. For instance, Das et al. [52] investigated a solution to learn the activity patterns of older adults and to detect errors. The authors trained a one-class SVM on raw sensor data and used an outlier detection algorithm to capture deviations in activities and prompt users to complete them. The solution showed the potential to assist medical personnel to capture the functional decline caused by dementia. Schinle et al. [54] used a set of activity indicators such as wake-up time, bed time, and night time to create a personalised profile for dementia patients. The profile served to enhance existing monitoring systems used by caregivers for ambulatory care. The authors applied the local outlier factor (LOF) clustering algorithm to distinguish between normal events and abnormal events reflecting signs of dementia.

Several smart home solutions have also been used to investigate behavioural differences between HOAs and those with cognitive impairment. Akl et al. [48] proposed a method to detect cognitive decline among HOAs and MCI patients based on location presence. The authors used inhomogeneous Poisson processes to build statistical models of users’ daily presence in different locations. The resulting generalised linear models (GLMs) were combined with Kullbak–Leibler (KL) divergence measures to distinguish between activity models representing HOAs and those with cognitive impairment. MCI was detected with an average area under the ROC curve of 0.716 and average area under the precision-recall curve of 0.706. The approach in [48] was extended by Akl et al. [49] to detect cognitive decline from activities performed by MCI, a-MCI, and non-amnestic (na-MCI) users. They clustered the distributions of walking and presence in the smart home using k-means and affinity propagation and classified them into HOAs and MCI patients. The solution was able to detect MCI patients with an F0.5 score of 0.856 and an F 0.5 score of 0.958 for na-MCI. Similarly, Ahamed et al. [58] proposed a method to capture early signs of dementia among HOAs and cognitive impaired users. The authors extracted various features (e.g., the number of completed tasks) from the CASAS dataset [31], and used them to train several classification models to identify cognitive impairment. The findings showed that the fine decision tree, fine KNN, ensemble boosted, and RUSBoosted methods perform better in identifying the onset of dementia.

Some solutions have focused on capturing early signs of dementia from simulated abnormal behaviour. For instance, Arifoglu and Bouchachia [53] added instances of forgotten and repeated activities to the Kasteren dataset [33]. They used the original data for the training of a model to learn normal behaviour and the generated data to learn abnormal behaviour. The authors trained variants of recurrent neural networks such as Vanilla RNNs and long short-term memory (LSTM), using features (sensor activation, change-point, and last-fired) to learn activity patterns and detect deviations. The results showed that RNNs outperformed most traditional techniques in recognising activities and detecting anomalies. In a subsequent study [56], the authors injected similar abnormal behaviours into two CASAS datasets (Aruba [31] and WSU [77]). The sensor data were mapped to the last-activated events, and input to convolutional neural networks (CNNs) to learn the encoding. They combined LSTM with CNNs to capture activity sequences representing user behaviour. The approach performed better at recognising activities than other techniques, such as NB and hidden Markov models (HMMs), and showed the potential to detect abnormal behaviour.

Similarly, Arifoglu et al. [57] analysed data on the Aruba dataset [31] containing the same simulated behaviour to capture anomalies indicating cognitive decline. They first trained graph convolutional networks (GCNs) on the original data and then deployed them on the data with simulated behaviour. Abnormal behaviours related to dementia were detected using confidence probabilities. Unlike in [53], the method did not consider temporal information or the relationship between time instances. The method enabled the recognition of activities and the detection of anomalies related to cognitive decline. Using the same dataset, Arifoglu et al. [30] proposed a solution to detect anomalies that indicate early signs of dementia. The activities were modelled using recursive auto-encoders (RAEs) to learn normal activities, whereas a RAE reconstruction error was computed to distinguish between normal and abnormal activity. Abnormal behaviours were abstracted at both the activity level and the sub-activity level. The method was compared with other approach, such as RNNS (LSTM variants) and CNNs, and showed better results when data were unlabelled.

Other studies used predefined rules to model contextual knowledge about the user’s activities in a smart home to enable reasoning. An example is the work of Gayathri et al. [50], who captured the abnormal behaviour of HOAs when performing activities such as making food. They used Markov logic network (MLN) to handle uncertainties from incomplete actions and to allow context reasoning. Features such as activity time and duration were organised hierarchically to facilitate layer-wise computation to speed up decision making. The method showed better accuracy at detecting abnormal behaviour than approaches such as the hidden Markov model (HMM).

Similarly, Riboni et al. [47] proposed a rule-based system to capture detailed information on the abnormal behaviour of MCI patients. The system inferred sensor events and actions through a rule-based inference engine and fed them to an MLN to deduce the activities. They modelled abnormal behaviours using domain knowledge and applied supervised learning to learn the rules matching sensor events to activity intervals. They also captured fine-grained abnormal behaviours through an inference engine and communicated alerts to caregivers for decision making. The system was evaluated by clinicians [78], and showed its potential to assist in diagnosing MCI. Riboni et al. [51] extended the previous study [47] by combining rules and machine learning to detect the abnormal behaviour of HOAs and an MCI patient. Contrary to communicating the inferred events and actions to MLN [47], the authors input them to a temporal feature model and then classified them into activities using RF. Next, they applied a SmartAggregation algorithm to deduce current activity instances based on predefined rules to provide personalised alerts to caregivers upon the detection of anomalies. The method performed better at recognising activities and detecting anomalies than other techniques, such as HMM.

Other solutions combined AR and AnD to provide automated health assessments. For instance, Alberdi et al. [55] monitored the activities of HOAs and MCI patients to predict health assessment scores (mobility, cognition, and mood) associated with early signs of AD. Features such as repeated activities were mapped to health assessment scores representing early signs of dementia. The authors applied several regression models to predict symptoms of AD, and classifiers such as an SVM to detect changes in the scores. The results showed the solution’s potential to detect early signs of AD.

The above methods included users not yet diagnosed with dementia. The methods presented in the next section were tested for users between pre-dementia and middle stage dementia.

### 4.3. Activity Recognition for Pre-Dementia to Middle Stage Dementia

Similarly to the previous sections, solutions for capturing behavioural differences between HOAs and those with cognitive impairment have also been investigated for pre-dementia and middle-stage dementia. For instance, Vuong et al. [59] used sensor data to recognise walking activity and movement patterns to detect the wandering patterns of healthy and dementia patients in a smart home. The authors applied a classification algorithm to classify the movements based on the wandering patterns via MS (direct, pacing, lapping, random). The algorithm was compared with dynamic time warping (DTW) and symbolic aggregate approximation and was found to produce better classification results. Urwyler et al. [61] also investigated the use of AR methods to capture differences in activity performance between HOAs and dementia patients. First, they used a pattern-recognition-based circadian activity rhythm (CAR) classifier to analyse sensor data. Then, they applied activity maps to visualise activity patterns and changes in these patterns. Activity performance, compared between the healthy subjects and those with dementia, was quantified with the help of a Poincaré plot (PP). The results showed more heterogeneity in the activity patterns performed by OAwDs than in those of HOAs. Similarly, Sprint et al. [62] investigated a method to recognise the activities of healthy and MCI or dementia patients and detect behavioural changes between the groups. Several features, such as the first and last activity instance, were fed to a change-point detection algorithm to determine the effect of impairment on the activity patterns. The method was able to detect multiple differences in activity patterns and walking speed between MCI and dementia patients, compared to healthy users.

As mentioned previously, modelling the context of the users’ activities in smart homes using predefined rules enables intelligent reasoning. Meditskos and Kompatsiaris [60] proposed a method to recognise activities performed by MCI and dementia patients in a smart home. The authors used ontologies to model associations between low-level activities and high-level activities. They used a context-aware algorithm and SPARQL rules to infer activities. The authors also implemented a telicity layer on top of the activity recognition module to capture interleaved activities for situational interpretation. The method performed well in recognising activities when compared with other methods such as HMM.

### 4.4. Anomaly Detection for Pre-Dementia to Middle-Stage Dementia

In this section, we discuss anomaly detection methods for users between pre-dementia and middle-stage dementia.

A few studies have applied user-defined rules to model the smart home context to reason about abnormal situations. For instance, Bouchard et al. [63] recognised the activities of HOAs and AD patients based on the layout of their homes and sensor data. The authors used predefined rules containing spatio-temporal constraints to model the activities. Spatial information such as position, distance, and orientation were analysed using qualitative spatial reasoning to detect the abnormal behaviours of AD patients. The solution was validated with clinical data and performed better at recognising activities and abnormal situations than other algorithms. In a similar study, Meditskos et al. [66] investigated a method to recognise the activities of OAwDs and to capture fine-grained abnormal behaviour based on the users’ profiles. The authors defined activities and users’ profiles using semantic web technologies (OWL/RDF) and represented them in knowledge graphs to infer contextual information. Features extracted from sensor data were classified using an SVM and a score was assigned to each activity. To detect abnormal situations, SPARQL queries were executed on the activity graphs, taking into account clinical information and user profiles. The solution was able to extract detailed contextual information about the users’ activities and abnormal situations. The information was visualised by clinicians and users for decision-making and personal choices, respectively.

Capturing behavioural differences between HOAs and those with cognitive impairment remains a focus of many activity recognition and anomaly detection methods. Alam et al. [64] proposed a method to analyse the activity performance and physiological data of MCI and dementia patients to distinguish between the two groups. They applied an SVM to obtain the activity scores automatically. Activity performance scores based on task completion, duration, and order in which activities were performed, were found to correlate with clinically observed scores. The results showed that cognitively healthy users completed their activities with less difficulty than those with dementia.

In a similar study, Varatharajan et al. [65] used wearable sensor data to recognise movement shapes of HOAs and AD patients to capture early signs of dementia. They classified gait signals of HOA and AD patients using a dynamic time warping (DTW) algorithm and compared them using the middle level cross identification (MidCross) algorithm. Observable differences in the walking speeds of healthy and patients with AD were noticed over time. The results showed that DTW outperforms other methods such as KNN and SVM. Chikhaoui et al. [68] also investigated a method to detect behavioural differences among healthy, MCI, and AD patients based on activity performance. The authors used two different sensor datasets containing activities such as preparing meals and making phone calls. Data features were extracted and classified using RF to capture the behavioural patterns of each group of users and detect anomalies in activity performance. On one dataset, the method accurately distinguished HOAs from users with cognitive impairment, but was less accurate on the dataset with fewer activity monitoring sensors. Similarly, Khodabandehloo and Riboni [67] proposed a solution to distinguish wandering from the movement trajectories of healthy, MCI, and AD patients captured on the CASAS dataset [79]. Unlike previous approaches to wandering, this study combined the home layout with the user’s trajectory to train a personalised model. New wandering events based on a clinical model were classified into episodes using RF. Episodes representing normal, possibly abnormal, and abnormal were classified as healthy, MCI, and AD, respectively. Long-term trajectory analysis showed that the method can detect cognitive decline and assist clinicians.

### 4.5. Activity Recognition for Early- to Middle-Stage Dementia

In this section, we discuss activity recognition methods that focus on context-based reasoning and behavioural differences for users from early-stage to middle-stage dementia.

Similarly to the previous sections, several methods used predefined rules to model users’ activities in smart homes to enable intelligent reasoning. Stavropoulos et al. [69] proposed a system to infer activities and provide personalised support for dementia patients. In addition to activity monitoring, the method was also used to analyse speech captured via microphones. Ontologies were used for the semantic integration of sensor data, domain knowledge, and users’ profiles. Using data analysis, the system was able to distinguish between healthy subjects, and those with early-age and late-stage dementia. The system demonstrated its usefulness in lab assessments and residential homes. Karakostas1 et al. [70] also proposed a holistic system for supporting an OAwD in performing daily activities. Semantic web technologies were applied to integrate and analyse multi-sensor data to recognise user activities. The sensor data were correlated and aggregated using SPARQL queries to infer meaningful information for the clinicians. The analysis provided clinicians with information to determine the status of the patient to make further decisions. The system improved the patient’s quality of life in several areas, including better sleep and improved personal hygiene.

A similar system based on machine learning techniques was proposed by Su et al. [71] to improve the independence of an OAwD in a smart home. The authors applied RF and finite state machine (FSM) classifiers to model the user’s position, whereas hand movements were captured using a Dirichlet process mixture model (DPMM) clustering algorithm. The position and hand movements were integrated using a dynamic Bayesian network (DBN) to obtain the activity patterns. The method was evaluated and showed 96.8% and 96.7% for precision and recall, with the potential to capture behavioural patterns and anomalies.

### 4.6. Anomaly Detection for Early- to Middle-Stage Dementia

The anomaly detection studies presented in this section either focus on context-based reasoning, anomaly prediction, or health assessments for users between the early stage and the middle stage of dementia.

A few methods have investigated solutions to represent smart home contexts using predefined rules to enable automated reasoning. For instance, Lazarou et al. [18] proposed a system to recognise the activities of MCI and dementia patients and provide personalised assistance with cognitive function, daily activities, and quality of life. The authors used an ontology to semantically integrate multi-sensor data and rules to infer activities and detect abnormal behaviour. The system was tested and showed its effectiveness in assisting users in their daily activities and supporting clinicians with neuropsychological assessments. In a similar study, Gayathri and Easwarakumar [73] proposed a decision support system to monitor the activities of a dementia patient and capture deviations from regular activities. The authors applied MLN on weighted features (location, time, and activity duration) and domain knowledge from caregivers or doctors to detect anomalies and trigger alerts. The system was designed to assist the patient with completing activity routines. The results showed a higher F-score measure compared to other approaches.

Other studies used activity patterns extracted from sensor data to predict anomalies. For instance, Lotfi et al. [72] developed a solution to recognise the activities of an elderly person with dementia and to detect and predict abnormal behaviour. They clustered the duration and start time of movement and room occupancy using different algorithms such as K-means and fuzzy C-means (FCM). Large and smaller clusters represented normal and abnormal activities, respectively. The authors applied a recurrent neural network (echo state network (ESN)) to predict abnormal activities and inform the caregivers when further assistance was required. The approach was reported to perform better than other techniques such as back-propagation through time (BPTT) and real-time recurrent learning (RTRL). In a similar study, Chalmers et al. [75] proposed a method to capture the behavioural patterns of OAwDs and predict abnormal behaviour. Unlike other approaches, the study only used smart meters to monitor electricity usage from home devices. The data were disaggregated and then used to extract three main features (min, max, and standard deviation). The authors applied SVM and RF models to classify the features for capturing behaviour patterns, and a Z-score to detect anomalies in device usage. The system was tested in a clinical trial and showed the potential to identify behavioural patterns, detect anomalies, and capture early signs of cognitive decline.

Apart from recognising activities and detecting anomalies, other researchers have also investigated methods to assess the health status of users. For instance, Enshaeifar et al. [36] proposed a solution to recognise activity patterns and detect changes in the routines of an OAwD. The authors used Markov chain and entropy rate analysis to extract high-level activity patterns and detect deviations from normal activity sequences, whereas K-means clustering was used for data analysis. They used hierarchical information fusion to detect signs of agitation, irritation, and aggression. The solution was able to recognise agitation and changes in activity patterns with high accuracy and was useful for diagnosis and support by medical professionals and clinicians. Similarly, Enshaeifar et al. [74] investigated a method to recognise changes in activity (sleep and movement) patterns to detect early symptoms of cognitive decline in dementia patients. The authors used non-negative matrix factorisation (NMF) to extract features from physiological data and cluster them to identify urinary tract infections. They also applied the isolation forest (iForest) method to analyse both physiological and environmental data to capture activity patterns and changes in patterns associated with early signs of dementia. The results showed that the NMF model performed better than traditional techniques and reduced the number of false alerts.

### 4.7. Activity Recognition for Late-Stage Dementia

We could not find activity recognition or anomaly detection methods addressing specific characteristics of OAwDs in late-stage dementia. The lack of studies may also be supported by the GDS assessment [15] presented in Section 2, which indicates that late-stage dementia is characterised by the patients’ reliance on caregivers to perform the majority of their daily activities. For this reason, current smart home technologies are most likely not supportive at this stage.

## 5. Habit Recognition for Older Adults with Dementia

As discussed throughout this paper, the state of the art of smart homes for OAwDs mainly focuses on activity recognition and anomaly detection. However, as previously argued, such techniques lack the capability to capture patients’ transitions to later stages of dementia. This capability would not only enable smart homes to adapt to their users throughout the evolution of their disease; it would also transform smart homes into invaluable diagnostic tools for doctors. In this section, we discuss how habit recognition could enable smart homes of the future to detect deterioration in OAwDs.

A habit is defined as one or more activities performed repeatedly in a similar manner [80]. Thus, to define a set of activities as a habit, there should be no or very little variation in the way that such activities are performed. An example of a habit in the context of dementia is *taking a donepezil pill with a glass of water every evening before going to bed* to control memory disturbances and improve cognitive function.

Consider the persona known as Frida, affected by middle-stage dementia, presented in Section 2. When the sun goes down, she starts getting anxious and performs strange activities, such as taking her clothes out of the closet. When transitioning from early-stage dementia to middle-stage dementia, patients may develop the habit of opening drawers and cabinets, as well as pacing back and forth when it gets dark. Patients might also try to ‘go home’, even when already at home, due to the fact that the home environment appears different from the one they imagine (e.g., a childhood home). Such behaviour, known as sundowning [81], is triggered by confusion, anxiety, and disorientation. Sundowning is a typical symptom in patients affected by middle-stage dementia, and its correlation with outside light conditions makes it interpretable as a habit.

As previously discussed, Moyle et al. [13] argue that current smart home solutions are not adequate to support OAwDs. The reason for their inadequacy is that current solutions implement solely AR and AnD routines. In the scope of AR and AnD, sundowning is only identified as an anomaly, a deviation from the normal routine of watching TV after taking a nap. In other words, AR and AnD are unable to detect the fact that sundowning is the new norm for Frida, which arose when she transitioned to middle-stage dementia. From this perspective, habit recognition becomes necessary.

A few studies have paved the way towards habit recognition in smart homes, such as those of Meng et al. [82], Lee and Melo [83], and Wang et al. [84]), but none of them were developed with the OAwDs in mind. Generic habit recognition systems would allow detection of the fact that a new habit has arisen (e.g., pacing at sunset), but they lack the capability of interpreting such changes in terms of transitions to different dementia stages. Again taking into consideration the persona designated as Frida, her wandering habit occurs in late afternoon, when it gets dark. A habit recognition system for OAwDs should not only capture new wandering habits, but also take into account at which time these habits take place. Wandering at sunset could be a stronger indicator of transitioning to middle-stage dementia than wandering in the early morning.

Apart from capturing habits and changes in habits, smart homes for OAwDs should be able to recognise the loss of habits. Losing some habits might indicate that the patients are transitioning to later stages of dementia. The capability of detecting such losses could be a critical feature for smart homes targeting dementia patients, as it could provide vital information to medical personnel. For instance, patients in the late stage of dementia are easily distressed by noise and they lose the ability to process complex information. As a consequence of these symptoms, the habit of *watching TV after taking a nap* that they exhibited during their middle-stage dementia may disappear. Generic habit recognition solutions lack the capability of putting this information, concerning *a patient who stops watching TV*, into the specific context of dementia symptomatology. Habit recognition tailored for dementia patients should be developed in order to overcome such limitations.

### Challenges for Implementing Habit Recognition in Smart Homes for OAwDs

The realisation of habit recognition solutions for OAwDs in smart homes faces challenges. There is a lack of publicly accessible real-world datasets for dementia patients. At the same time, acquiring annotated datasets of OAwDs for the evaluation of habit recognition solutions is challenging, given the ethical and privacy issues. To compensate for the scarcity of realistic datasets, some studies have [53,57] generated artificial data by injecting non-patient datasets with activities that reflect the conditions of OAwDs, e.g., forgetting or repeating activities.

Pattern recognition methods (e.g., symbolic aggregate approximation (SAX) [85]) and unsupervised machine learning algorithms (e.g., LSTM [86]) can be alternative solutions to the lack of annotated data discussed above. On the one hand, this saves the domain experts from manually labelling an extensive amount of data, which is normally required in order to implement supervised learning approaches. On the other hand, applying these methods to learn the habits of users and detect changes related to the transitions of dementia stages requires large volumes of unlabelled data for training. These data may need to be collected over long periods according to the duration of each stage of dementia. For instance, the duration of MCI is estimated to last on average for 3.1 years, whereas mild-stage dementia, moderate-stage dementia, and severe-stage dementia last for 3.5 years, 2.0 years, and 1.3 years, respectively [87].

Furthermore, dementia is complex, which makes it difficult to learn the habits of the patients. Each patient behaves differently, with some users experiencing a gradual decline, whereas others may show a sudden and unpredictable decline [88]. Some symptoms of dementia may also overlap between different stages [22], making it difficult to associate habits with specific dementia stages.

Due to the unpredictable nature of dementia, it is challenging to acquire expert knowledge regarding the features that are important for recognising habits and changes in habits of OAwDs. Chimamiwa et al. [89] proposed the extraction of features (duration and frequency) for habit recognition from the activities of a healthy user. However, the features were chosen arbitrarily without considering the habits of dementia patients.

Integrating pattern recognition methods with knowledge-based methods could enable the extraction of features based on how an individual’s habits evolve over time [89]. In this way, the changes that are captured can be used to automatically update the domain knowledge with new information, thereby freeing the human developer from the cumbersome task of manually modelling every detail as to how an individual performs activities.

Another challenge is the ability to reconcile the symptoms of OAwDs with multi-occupancy environments. Howedi et al. [90] showed that entropy measures of motion sensors can be used for determining the presence of multiple users. However, as previously mentioned, OAwDs may act randomly, depending on the severity of the disease, which could lead to high entropy and could be incorrectly interpreted as multi-occupancy. Giaretta and Loutfi [91] proved that it is possible to map a smart home to an undirected graph and list the scenarios that can only be explained by multi-occupancy.

Another issue is that, as patients move to later stages of dementia, caregivers may need to be always present to monitor and care for the OAwDs. This entails the problem of discerning the actions of the OAwDs from the actions of their caregivers. In smart homes, this problem can be addressed by monitoring users with wearable RFID tags that uniquely identify each individual [92]. However, such techniques may not be effective, given that OAwDs may often forget to wear the sensors. Further investigations are needed to address this obstacle.

## 6. Related Works

Several review studies have been conducted on smart home technologies for older adults with dementia. Moyle et al. [13] conducted a comprehensive review on the effectiveness of smart homes to support the health outcomes of community-dwelling OAwDs. The authors evaluated the effectiveness of smart home systems in measuring physical activity, activities of daily living, sleep, anxiety, depression, agitation, irritability, the risk of falls, cognitive functioning, night-time injury, and unattended home exits. They concluded that current smart home systems lack a comprehensive evaluation due to the poor quality in their methodology and reporting. However, the authors did not consider how the various aspects relate to specific stages of dementia. In our study, we categorise the smart home technologies based on the stage of dementia, which could serve to highlight the progress of smart homes towards assisting not only early-stage patients, but also those at later stages.

Another literature review conducted by Zamiri et al. [93] classified smart home technologies and services based on their functionalities, capabilities, and features. The purpose of the study was to evaluate the use of smart home technologies to improve the quality of life of OAwDs, based on how easily these technologies could be customised. The authors then proposed a range of categories for classifying smart home technologies, namely, cognitive, environmental, functional, tele-information, physiological, and social. The cognitive category addressed the health functions associated with dementia, such as thinking, perception, memory, and problem solving. However, the study did not match the functions against specific stages of dementia, whereas in our study we provide detailed discussions on the various symptoms of dementia at each stage of the disease. In addition, we highlight relevant existing technologies for assisting patients at each stage.

Finally, the literature review conducted by van Boekel et al. [94] focused on the perspectives of stakeholders regarding the use of technology by people with dementia. They found that most perspectives on the use of technology for OAwDs were provided by informal caregivers and less by formal caregivers or dementia patients. As we discuss in this study, the use of personas might be useful in the development of future smart homes for older adults with dementia, as they serve to provide information about the actual challenges experienced by patients. Furthermore, we argue that, given the complex nature of dementia, domain expert knowledge is important for developing effective smart homes for OAwDs, particularly for habit recognition and change detection.

## 7. Conclusions

Most smart home solutions for OAwDs focus on activity recognition and anomaly detection. As argued in this study, such techniques lack the capability to recognise users’ habits, changes in habits, and the loss of habits, which may indicate a transition in the dementia stage. In this paper, we presented an overview of the stages of dementia and discussed real-world personas that help to underline the challenges experienced by OAwDs at each stage. We used the stages to organise the state of the art on activity recognition and anomaly detection, taking into account the goal of each method and the targeted users. Of the studies found, none addressed late-stage dementia, as the main aim of smart homes for OAwDs is to delay the cognitive decline by recognising the dementia stage as early as possible. However, in the late stage, OAwDs depend on the caregiver for most of their daily activities. Finally, we discussed several challenges faced in developing habit recognition solutions for OAwDs in smart homes. These challenges include the unavailability of good and annotated data for OAwDs, the lack of knowledge on the habits of dementia patients, and the lack of novel solutions to detect multi-occupancy in the residences of OAwDs.

## Figures and Tables

**Table 1:** Summary of methods of AR for OAwDs.

Study	Goal	Dementia Stage	Users
[38,39,40,41,42,43,44,45,46]	AR	Pre-dementia	Healthy/MCI
[30,47,48,49,50,51,52,53,54,55,56,57,58]	AR + AnD	Pre-dementia	Healthy/MCI
[59,60,61,62]	AR	Pre-dementia/Early/Middle	Healthy/MCI/Dementia
[63,64,65,66,67,68]	AR + AnD	Pre-dementia/Early/Middle	Healthy/MCI/Dementia
[69,70,71]	AR	Early/Middle	Dementia
[18,36,72,73,74,75]	AR + AnD	Early/Middle	Dementia
—	—	Late	Dementia

## Data Availability

The study did not report any data.
